# Supplementary material for: The response to influenza vaccination is associated with DNA methylation-driven regulation of T cell innate antiviral pathways
Source: Clin Epigenetics. 2024 Aug 21;16:114. doi: 10.1186/s13148-024-01730-x (PMC11340180; doi:10.1186/s13148-024-01730-x)
Supplement: Supplementary file 1 — Additional file 1: Figure S1. A Age distribution of this cohort in 2019. B Association of age with UGA3 and UGA4 vaccine strain responsiveness in 2019. C Association of age with UGA4 and UGA5 vaccine strain responsiveness in 2020. Figure S2. A Correlation between model features of the multivariate multiple regression model. B Full coefficient p value heatmap of the model. C Overlay of Manhattan plots for four vaccine strains. Red dashed lines indicate the adjusted p value < 0.05 threshold. Figure S3. Histone mark and variants & chromatin accessibility enrichment from Cistrome. A Significant positive HAI coefficient for histone mark and variants. B Significant positive HAI coefficient for chromatin accessibility. C Significant negative HAI coefficient for histone mark and variants. D Significant negative HAI coefficient for chromatin accessibility. Figure S4. Transcription factors binding sites enrichment of age and BMI. A Significant positive coefficient for age. B Significant negative coefficient for age. C Significant positive coefficient for BMI. D Significant negative coefficient for BMI. Figure S5. Transcription factors binding sites enrichment of significant negative HAI coefficients. A Distance from significant negative HAI coefficient sites to transcription start sites percentage. B Significant transcription factor binding sites from the Cistrome database, specifically focusing on either blood or immune related sources. C Overlap between methylation site and publicly available ChIP-Seq data from LOLA. Figure S6. A Correlation between DNA methylation and gene expression between responders and non-responders at day 0 and day 28 of UGA5. Promoter regions are +/− 1000 base pairs from TSS. B An example gene TRIM47’s expression and its corresponding methylation site methylation level at day 0 and day 28. Figure S7. A Estimates of cell-type proportions from RNA expression. B Heatmap of coefficients for selected DNA methylation sites mapped to differentially express [file 13148_2024_1730_MOESM1_ESM.pdf]

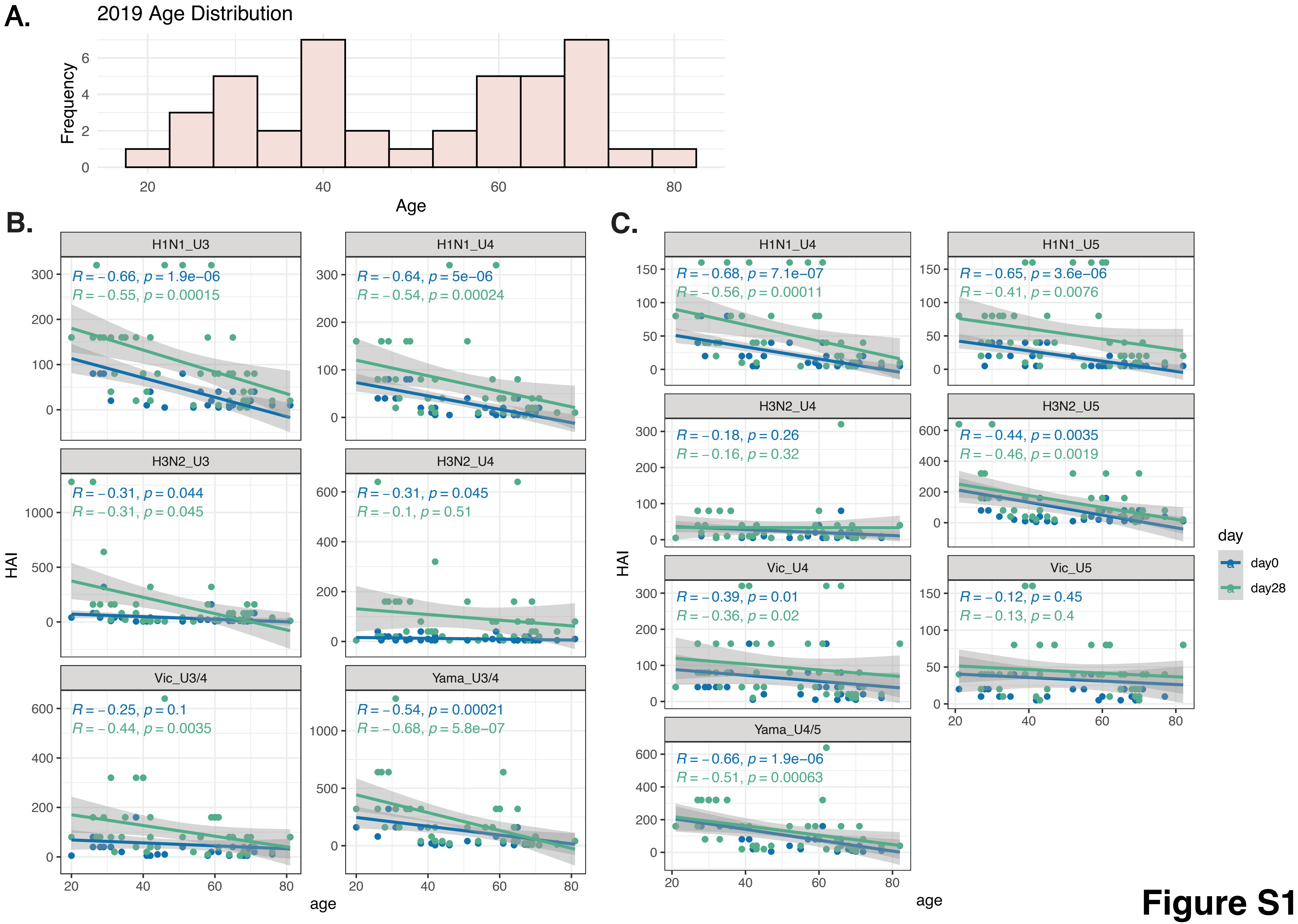
A.
2019 Age Distribution
Frequency
Age
B.
H1N1_U3
R = −0.66, p = 1.9e−06
R = −0.55, p = 0.00015
H1N1_U4
R = −0.64, p = 5e−06
R = −0.54, p = 0.00024
H3N2_U3
R = −0.31, p = 0.044
R = −0.31, p = 0.045
H3N2_U4
R = −0.31, p = 0.045
R = −0.1, p = 0.51
Vic_U3/4
R = −0.25, p = 0.1
R = −0.44, p = 0.0035
Yama_U3/4
R = −0.54, p = 0.00021
R = −0.68, p = 5.8e−07
HAI
age
C.
H1N1_U4
R = −0.68, p = 7.1e−07
R = −0.56, p = 0.00011
H1N1_U5
R = −0.65, p = 3.6e−06
R = −0.41, p = 0.0076
H3N2_U4
R = −0.18, p = 0.26
R = −0.16, p = 0.32
H3N2_U5
R = −0.44, p = 0.0035
R = −0.46, p = 0.0019
Vic_U4
R = −0.39, p = 0.01
R = −0.36, p = 0.02
Vic_U5
R = −0.12, p = 0.45
R = −0.13, p = 0.4
Yama_U4/5
R = −0.66, p = 1.9e−06
R = −0.51, p = 0.00063
HAI
age
day
day0
day28

Figure S1

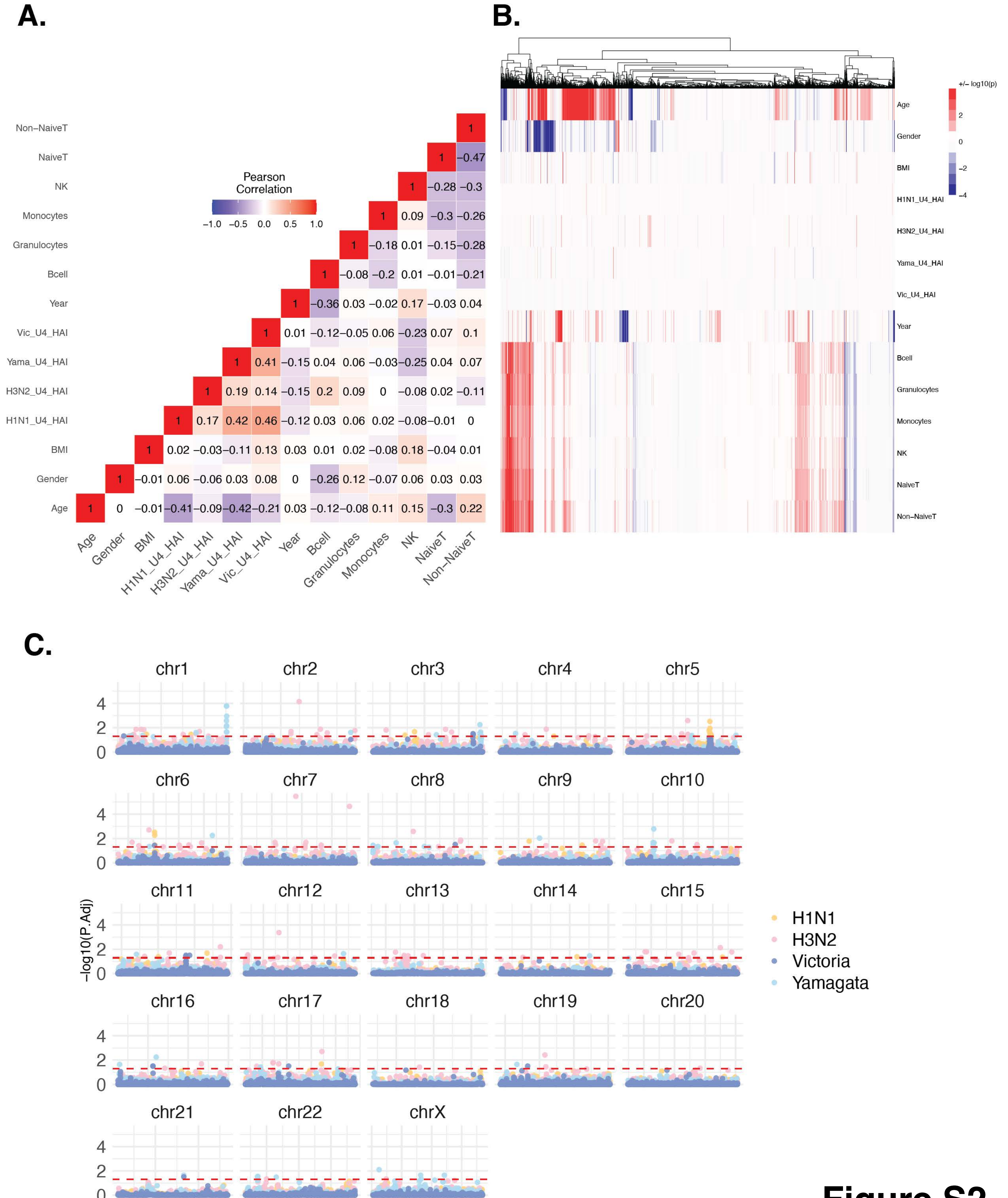

A.
Pearson Correlation
−1.0 −0.5 0.0 0.5 1.0
Age
Gender
BMI
H1N1_U4_HAI
H3N2_U4_HAI
Yama_U4_HAI
Vic_U4_HAI
Year
Bcell
Granulocytes
Monocytes
NK
NaiveT
Non−NaiveT
B.
+/− log10(p)
2
0
−2
−4
C.
chr1 chr2 chr3 chr4 chr5 chr6 chr7 chr8 chr9 chr10 chr11 chr12 chr13 chr14 chr15 chr16 chr17 chr18 chr19 chr20 chr21 chr22 chrX
−log10(P.Adj)
H1N1
H3N2
Victoria
Yamagata

Figure S2

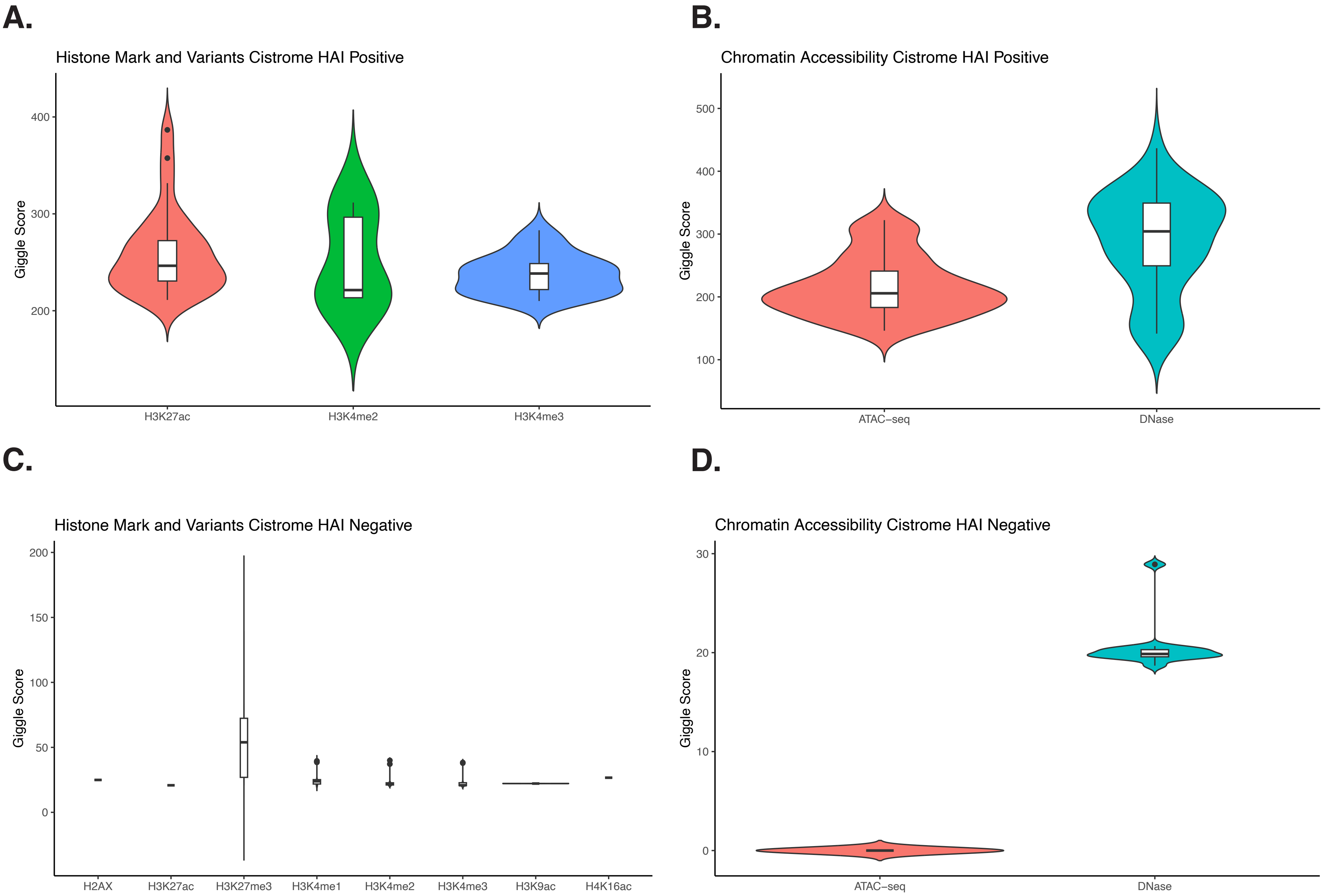
A.
Histone Mark and Variants Cistrome HAI Positive
Giggle Score
400
300
200
H3K27ac
H3K4me2
H3K4me3
B.
Chromatin Accessibility Cistrome HAI Positive
Giggle Score
500
400
300
200
100
ATAC−seq
DNase
C.
Histone Mark and Variants Cistrome HAI Negative
Giggle Score
200
150
100
50
0
H2AX
H3K27ac
H3K27me3
H3K4me1
H3K4me2
H3K4me3
H3K9ac
H4K16ac
D.
Chromatin Accessibility Cistrome HAI Negative
Giggle Score
30
20
10
0
ATAC−seq
DNase

Figure S3

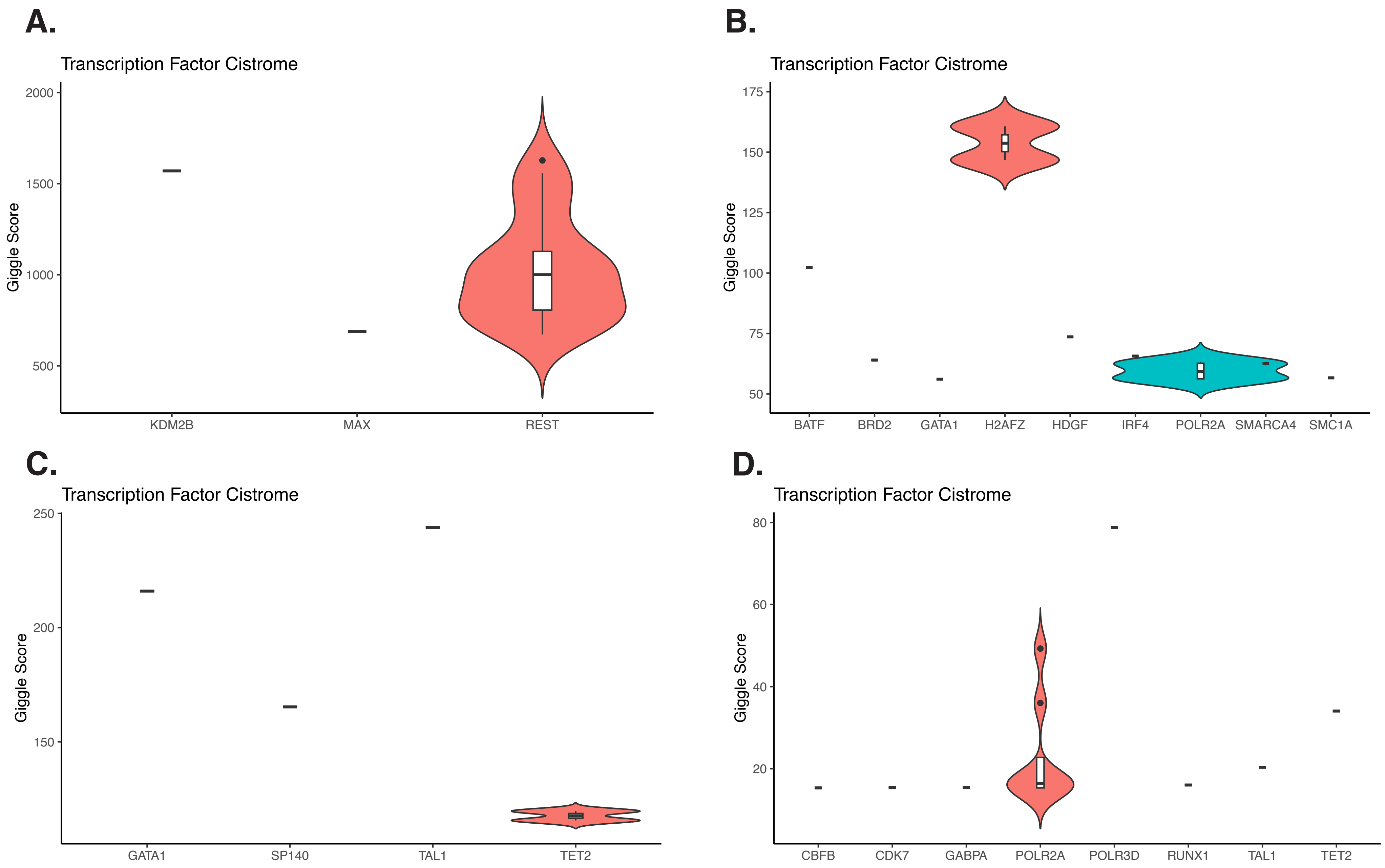
A.
Transcription Factor Cistrome
Giggle Score
2000
1500
1000
500
KDM2B
MAX
REST
B.
Transcription Factor Cistrome
Giggle Score
175
150
125
100
75
50
BATF
BRD2
GATA1
H2AFZ
HDGF
IRF4
POLR2A
SMARCA4
SMC1A
C.
Transcription Factor Cistrome
Giggle Score
250
200
150
GATA1
SP140
TAL1
TET2
D.
Transcription Factor Cistrome
Giggle Score
80
60
40
20
CBFB
CDK7
GABPA
POLR2A
POLR3D
RUNX1
TAL1
TET2

Figure S4

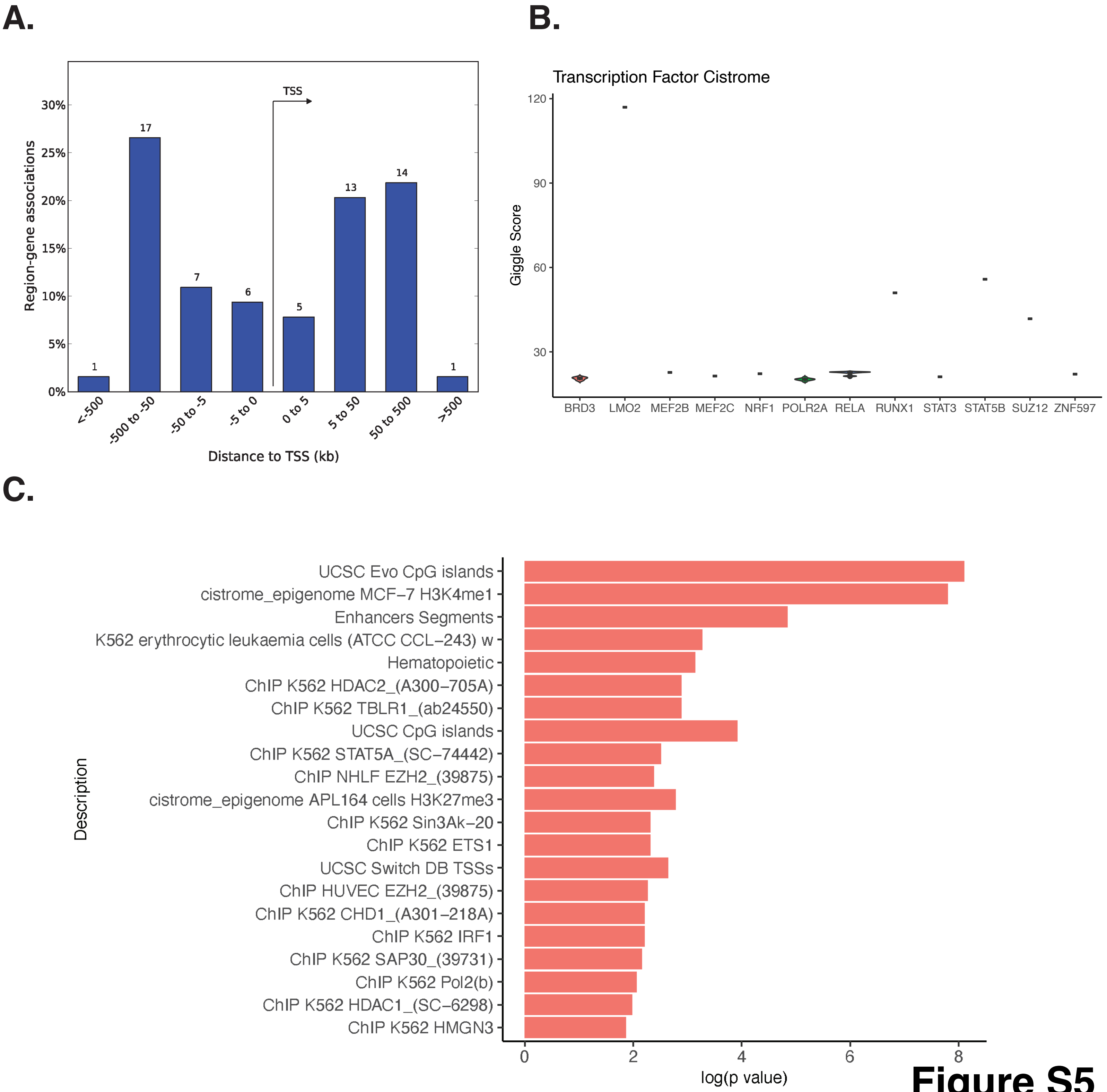

A.
B.
C.
TSS
Region-gene associations
Distance to TSS (kb)
<-500
-500 to -50
-50 to -5
-5 to 0
0 to 5
5 to 50
50 to 500
>500
Transcription Factor Cistrome
Giggle Score
BRD3
LMO2
MEF2B
MEF2C
NRF1
POLR2A
RELA
RUNX1
STAT3
STAT5B
SUZ12
ZNF597
Description
UCSC Evo CpG islands
cistrome_epigenome MCF−7 H3K4me1
Enhancers Segments
K562 erythrocytic leukaemia cells (ATCC CCL−243) w
Hematopoietic
ChIP K562 HDAC2_(A300−705A)
ChIP K562 TBLR1_(ab24550)
UCSC CpG islands
ChIP K562 STAT5A_(SC−74442)
ChIP NHLF EZH2_(39875)
cistrome_epigenome APL164 cells H3K27me3
ChIP K562 Sin3Ak−20
ChIP K562 ETS1
UCSC Switch DB TSSs
ChIP HUVEC EZH2_(39875)
ChIP K562 CHD1_(A301−218A)
ChIP K562 IRF1
ChIP K562 SAP30_(39731)
ChIP K562 Pol2(b)
ChIP K562 HDAC1_(SC−6298)
ChIP K562 HMGN3
log(p value)

Figure S5

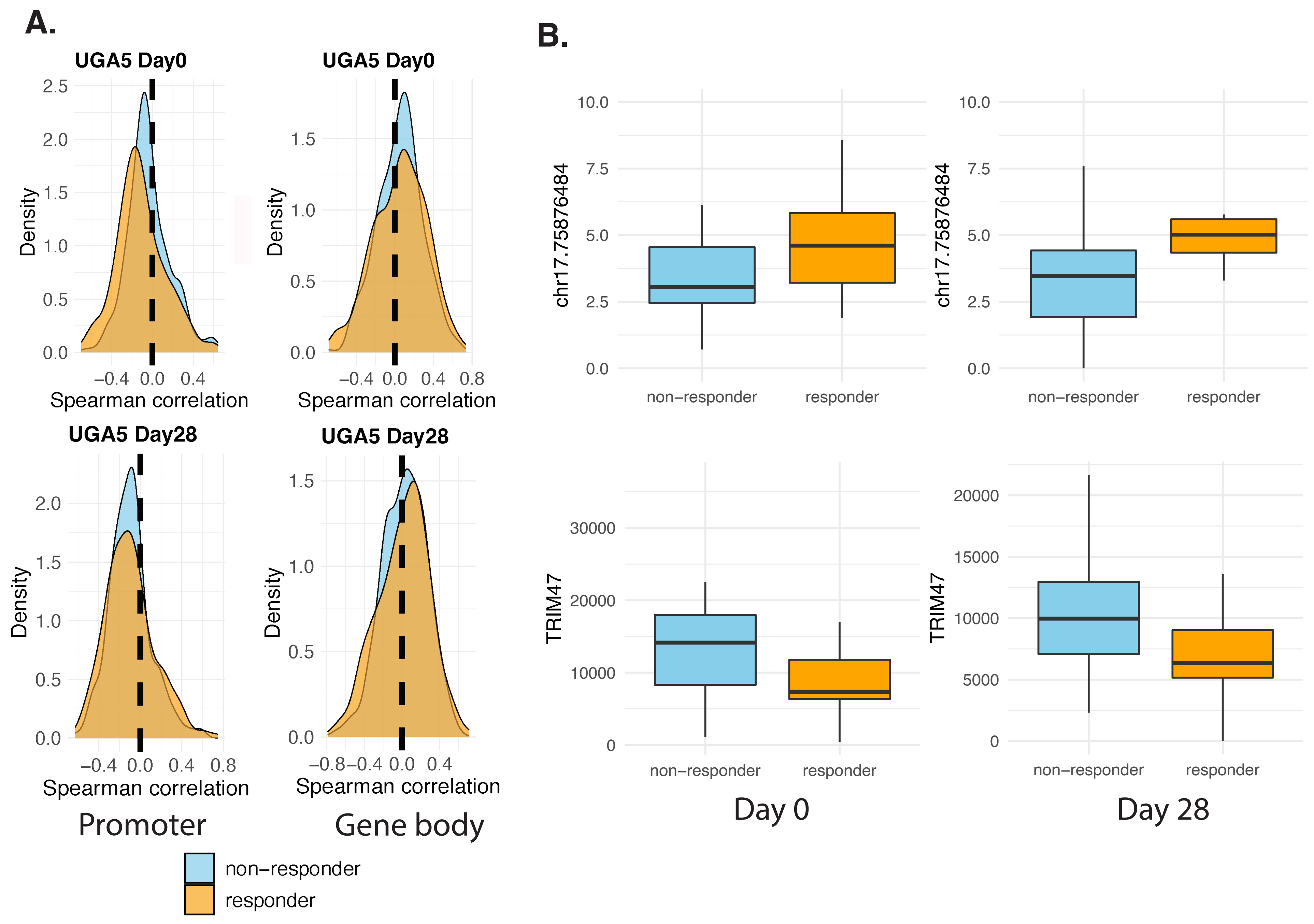
A.
UGA5 Day0
UGA5 Day0
Density
Spearman correlation
UGA5 Day28
UGA5 Day28
Promoter
Gene body
non−responder
responder
B.
chr17.75876484
TRIM47
non−responder
responder
Day 0
Day 28

Figure S6

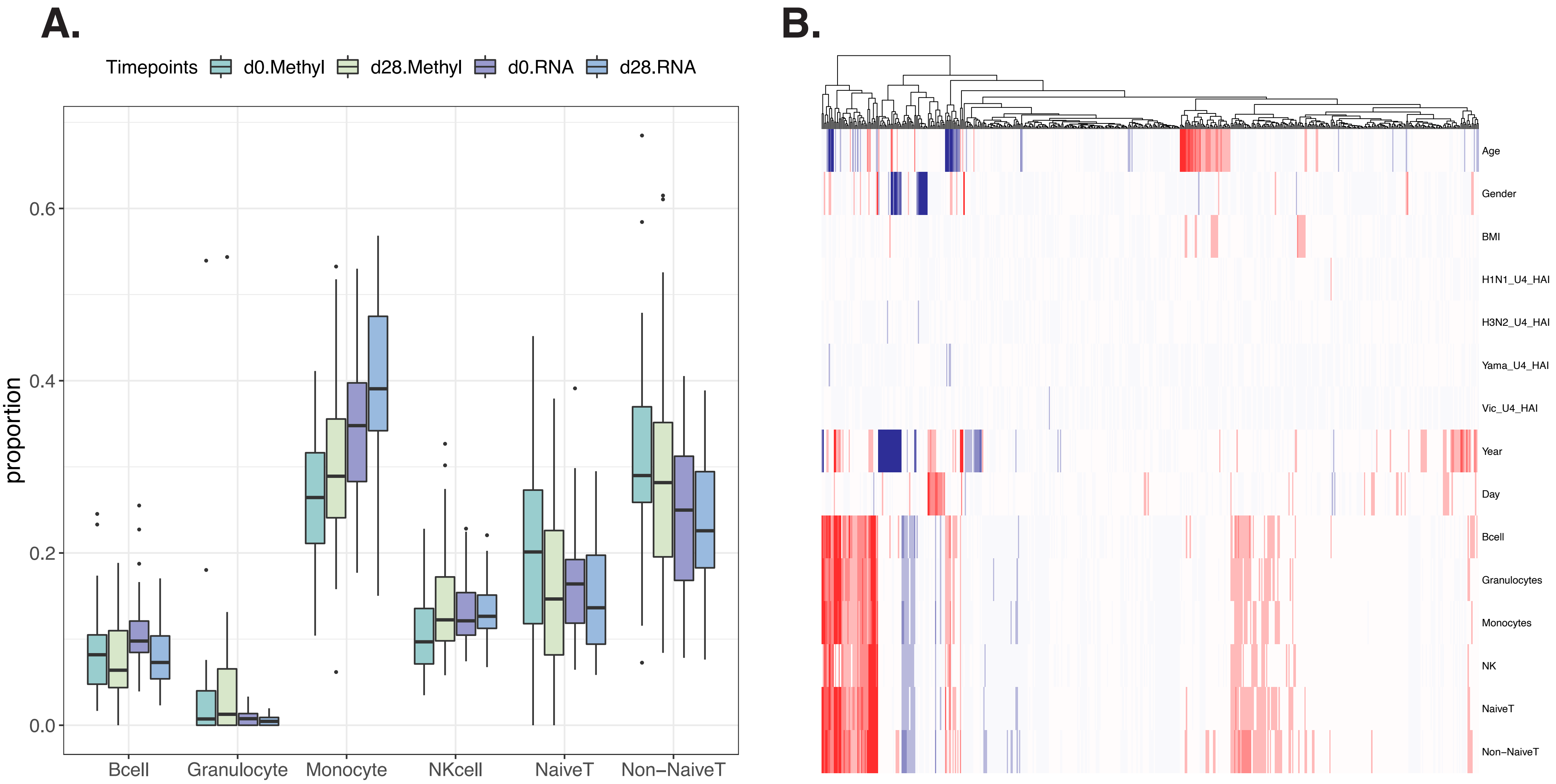
A.
Timepoints
d0.Methyl
d28.Methyl
d0.RNA
d28.RNA
proportion
0.0
0.2
0.4
0.6
Bcell
Granulocyte
Monocyte
NKcell
NaiveT
Non−NaiveT
B.
Age
Gender
BMI
H1N1_U4_HAI
H3N2_U4_HAI
Yama_U4_HAI
Vic_U4_HAI
Year
Day
Bcell
Granulocytes
Monocytes
NK
NaiveT
Non−NaiveT

Figure S7

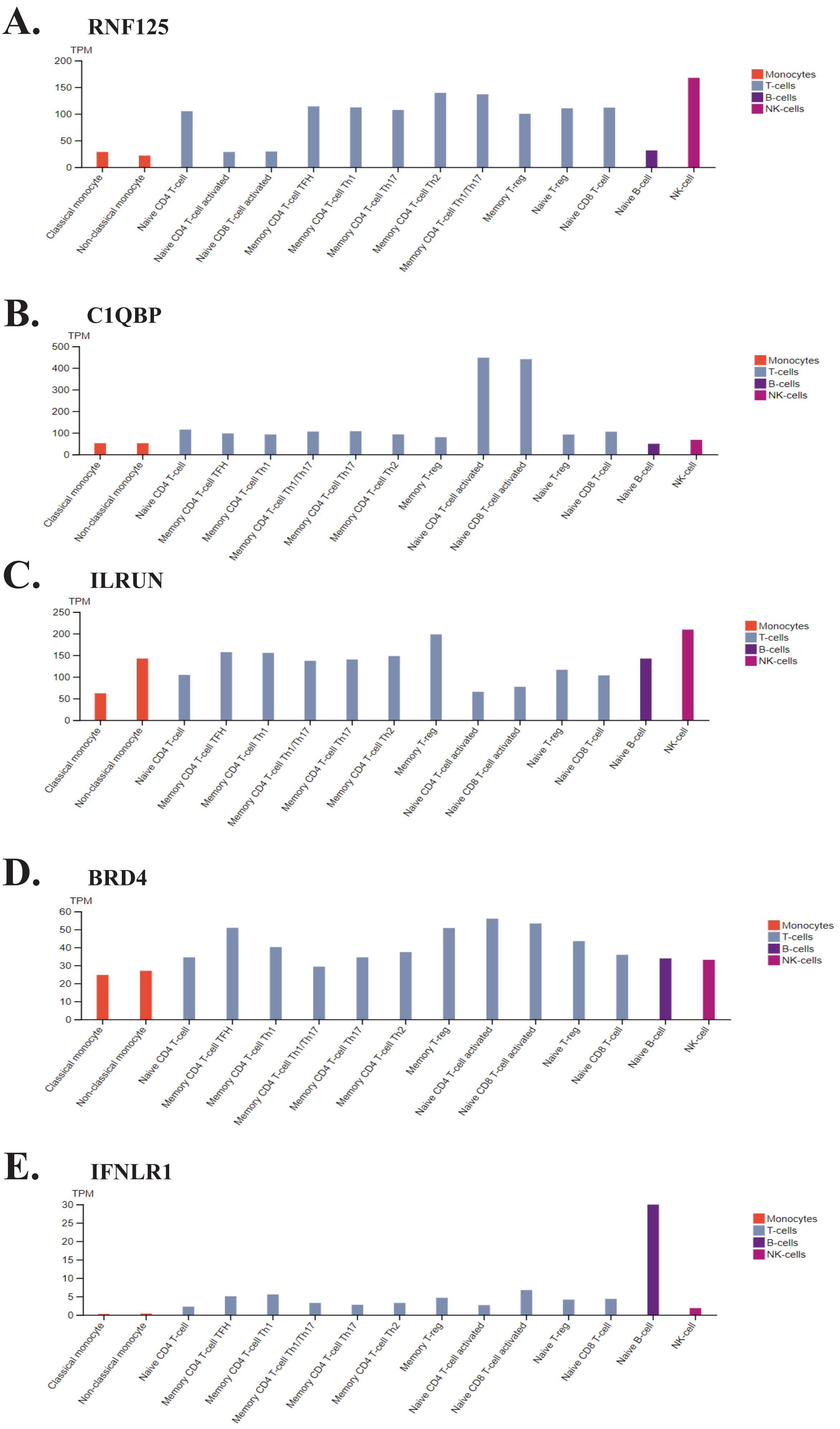
A.
RNF125
TPM
200
150
100
50
0
Classical monocyte
Non-classical monocyte
Naive CD4 T-cell
Naive CD4 T-cell activated
Naive CD8 T-cell activated
Memory CD4 T-cell TFH
Memory CD4 T-cell Th1
Memory CD4 T-cell Th17
Memory CD4 T-cell Th2
Memory CD4 T-cell Th1/Th17
Memory T-reg
Naive T-reg
Naive CD8 T-cell
Naive B-cell
NK-cell
Monocytes
T-cells
B-cells
NK-cells
B.
C1QBP
TPM
500
400
300
200
100
0
Classical monocyte
Non-classical monocyte
Naive CD4 T-cell
Memory CD4 T-cell TFH
Memory CD4 T-cell Th1
Memory CD4 T-cell Th1/Th17
Memory CD4 T-cell Th17
Memory CD4 T-cell Th2
Memory T-reg
Naive CD4 T-cell activated
Naive CD8 T-cell activated
Naive T-reg
Naive CD8 T-cell
Naive B-cell
NK-cell
Monocytes
T-cells
B-cells
NK-cells
C.
ILRUN
TPM
250
200
150
100
50
0
Classical monocyte
Non-classical monocyte
Naive CD4 T-cell
Memory CD4 T-cell TFH
Memory CD4 T-cell Th1
Memory CD4 T-cell Th1/Th17
Memory CD4 T-cell Th17
Memory CD4 T-cell Th2
Memory T-reg
Naive CD4 T-cell activated
Naive CD8 T-cell activated
Naive T-reg
Naive CD8 T-cell
Naive B-cell
NK-cell
Monocytes
T-cells
B-cells
NK-cells
D.
BRD4
TPM
60
50
40
30
20
10
0
Classical monocyte
Non-classical monocyte
Naive CD4 T-cell
Memory CD4 T-cell TFH
Memory CD4 T-cell Th1
Memory CD4 T-cell Th1/Th17
Memory CD4 T-cell Th17
Memory CD4 T-cell Th2
Memory T-reg
Naive CD4 T-cell activated
Naive CD8 T-cell activated
Naive T-reg
Naive CD8 T-cell
Naive B-cell
NK-cell
Monocytes
T-cells
B-cells
NK-cells
E.
IFNLR1
TPM
30
25
20
15
10
5
0
Classical monocyte
Non-classical monocyte
Naive CD4 T-cell
Memory CD4 T-cell TFH
Memory CD4 T-cell Th1
Memory CD4 T-cell Th1/Th17
Memory CD4 T-cell Th17
Memory CD4 T-cell Th2
Memory T-reg
Naive CD4 T-cell activated
Naive CD8 T-cell activated
Naive T-reg
Naive CD8 T-cell
Naive B-cell
NK-cell
Monocytes
T-cells
B-cells
NK-cells

Figure S8
