# Supplementary material for: The response to influenza vaccination is associated with DNA methylation-driven regulation of T cell innate antiviral pathways
Source: Clin Epigenetics. 2024 Aug 21;16:114. doi: 10.1186/s13148-024-01730-x (PMC11340180; doi:10.1186/s13148-024-01730-x)
Supplement: Supplementary file 3 — Additional file 3: Biorender publication license for Fig. 6. [file 13148_2024_1730_MOESM3_ESM.pdf]

49 Spadina Ave. Suite 200
Toronto ON M5V 2J1 Canada
www.biorender.com

Confirmation of Publication and Licensing Rights

April 8th, 2024
Science Suite Inc.

Subscription: *Student Plan*
Agreement number: *HS26O8WSY1*
Journal name: *Clinical Epigenetics*

To whom this may concern,

This document is to confirm that Hongxiang Fu has been granted a license to use the BioRender content, including icons, templates and other original artwork, appearing in the attached completed graphic pursuant to BioRender's Academic License Terms. This license permits BioRender content to be sublicensed for use in journal publications.

All rights and ownership of BioRender content are reserved by BioRender. All completed graphics must be accompanied by the following citation: "Created with BioRender.com".

BioRender content included in the completed graphic is not licensed for any commercial uses beyond publication in a journal. For any commercial use of this figure, users may, if allowed, recreate it in BioRender under an Industry BioRender Plan.

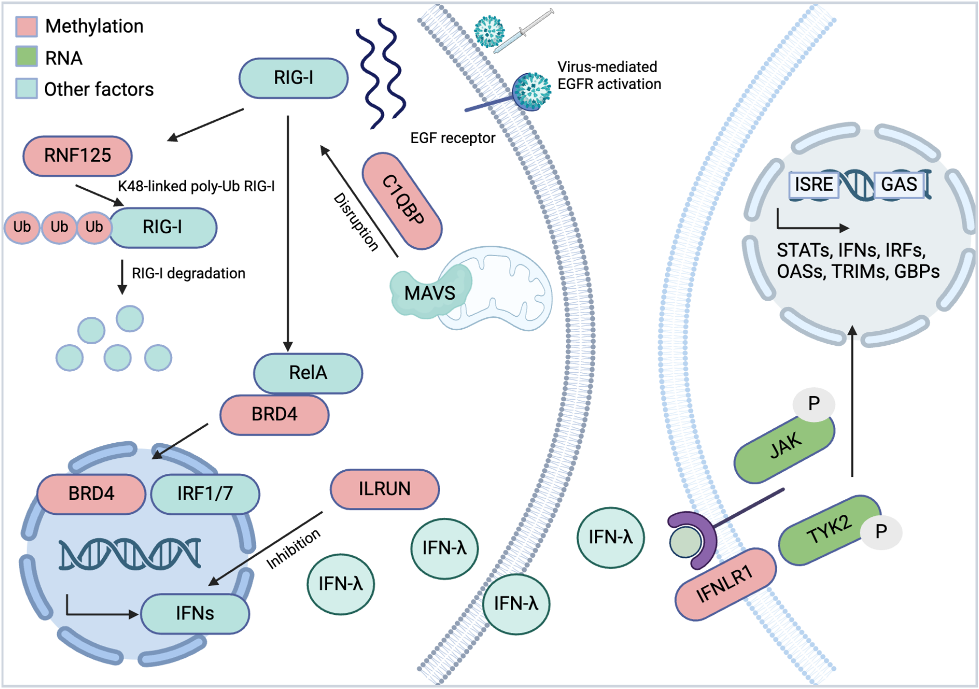

For any questions regarding this document, or other questions about publishing with BioRender refer to our BioRender Publication Guide, or contact BioRender Support at support@biorender.com.
